# New Insights on Dietary Polyphenols for the Management of Oxidative Stress and Neuroinflammation in Diabetic Retinopathy

**DOI:** 10.3390/antiox12061237

**Published:** 2023-06-08

**Authors:** Gustavo Bernardes Fanaro, Marcelo Rodrigues Marques, Karin da Costa Calaza, Rafael Brito, André Moreira Pessoni, Henrique Rocha Mendonça, Deborah Emanuelle de Albuquerque Lemos, José Luiz de Brito Alves, Evandro Leite de Souza, Marinaldo Pacífico Cavalcanti Neto

**Affiliations:** 1 Institute of Health and Biotechnology, Federal University of Amazonas, Manaus 69460000, Amazonas, Brazil; gbfanaro@ufam.edu.br; 2 Federal Institute of Science and Technology of Maranhão, Zé Doca 65365000, Maranhão, Brazil; marcelo.marques@ifma.edu.br; 3 Department of Neurobiology, Institute of Biology, Fluminense Federal University, Niterói 24210201, Rio de Janeiro, Brazil; kcalaza@id.uff.br; 4 Department of Cellular and Molecular Biology, Institute of Biology, Fluminense Federal University, Niterói 24210201, Rio de Janeiro, Brazil; rafaelbrito@id.uff.br; 5 Faculté de Medicine, Université Laval, Quebec, QC G1V 0A6, Canada; andre.moreira-pessoni.1@ulaval.ca; 6 Institute of Biodiversity and Sustainability (NUPEM), Federal University of Rio de Janeiro, Macaé 27965045, Rio de Janeiro, Brazil; hrmendonca@macae.ufrj.br (H.R.M.); m_neto_10@ufrj.br (M.P.C.N.); 7 Department of Nutrition, Health Sciences Center, Federal University of Paraíba, João Pessoa 58051900, Paraíba, Brazil; debralemooss@gmail.com (D.E.d.A.L.); jose.luiz@academico.ufpb.br (J.L.d.B.A.)

**Keywords:** diabetes, retina, polyphenols, cell death, antioxidant effects, neuroprotection

## Abstract

Diabetic retinopathy (DR) is a neurodegenerative and vascular pathology that is considered one of the leading causes of blindness worldwide, resulting from complications of advanced diabetes mellitus (DM). Current therapies consist of protocols aiming to alleviate the existing clinical signs associated with microvascular alterations limited to the advanced disease stages. In response to the low resolution and limitations of the DR treatment, there is an urgent need to develop more effective alternative therapies to optimize glycemic, vascular, and neuronal parameters, including the reduction in the cellular damage promoted by inflammation and oxidative stress. Recent evidence has shown that dietary polyphenols reduce oxidative and inflammatory parameters of various diseases by modulating multiple cell signaling pathways and gene expression, contributing to the improvement of several chronic diseases, including metabolic and neurodegenerative diseases. However, despite the growing evidence for the bioactivities of phenolic compounds, there is still a lack of data, especially from human studies, on the therapeutic potential of these substances. This review aims to comprehensively describe and clarify the effects of dietary phenolic compounds on the pathophysiological mechanisms involved in DR, especially those of oxidative and inflammatory nature, through evidence from experimental studies. Finally, the review highlights the potential of dietary phenolic compounds as a prophylactic and therapeutic strategy and the need for further clinical studies approaching the efficacy of these substances in DR management.

## 1. Introduction

Diabetic retinopathy (DR) is the most common complication of diabetes mellitus (DM) and the leading cause of blindness, affecting approximately 95% of patients with type 1 DM and more than 60% of patients with type 2 DM. DR is a public health and socioeconomic burden that could be prevented or reduced by implementing interventions, such as glycemic control, healthy lifestyle behaviors, and dietary modification [1,2,3].

According to data from the International Diabetes Federation (IDF) [4], 537 million people aged 20 to 79 years were diagnosed with diabetes worldwide in 2021, an increase of 16% over the previous two years, and projections for 2030 point to 643 million cases. The alarming global evolution of DM and the increasing development and progression of DR suggest early diagnosis and more effective, accessible, and cost-effective prophylactic and therapeutic measures.

Poor glycemic and blood pressure control and duration of diabetes are significant risk factors for developing DR, and rigorous control of these parameters promotes long-term benefits in delaying or preventing DR [5,6,7,8,9,10,11,12]. Pregnancy and puberty [13,14,15,16], kidney disease [17,18], and a high body mass index (BMI) [19] are also risk factors for DR. Even when these risk factors are under control, some patients still develop diabetic macular edema (DME), the major vision-threatening late complication of DR [20,21].

The nutritional profile is also a determining factor for DR [22,23]. The correlation between the consumption of different dietary components (macro- or micronutrients) [24,25,26] or even specific dietary patterns (e.g., Mediterranean diet) has been investigated in this context [27,28]. Despite the growing body of evidence linking dietary intake and DR, current data are inconclusive, for example, on which dietary components are associated with the protection or susceptibility to DR [22].

Although clinical protocols and nutritional recommendations are well established in treating DM, there is still a lack of specific dietary recommendations for DR [29,30]. In this sense, the development and implementation of specific nutritional protocols and therapies with bioactive compounds may represent an attractive, accessible, and cost-effective strategy in the management of DR [31,32,33].

Despite remarkable progress in understanding the molecular and pathophysiological mechanisms of DR, some gaps remain, including those related to the early stages of DR. Currently, therapeutic strategies, including glycemic and blood pressure control, laser photocoagulation, intravitreal injections of anti-inflammatory or VEGF neutralizing agents, and vitreoretinal surgery are limited to late stages of the disease, reducing the chances of preventing vision loss [34,35,36,37,38]. In addition, not all patients respond satisfactorily to current clinical therapies, suggesting that developing effective strategies for the prevention and treatment of DR is critical [36,38].

Polyphenols are a diverse group of bioactive molecules derived primarily from plant foods, such as fruits, vegetables, whole grains, coffee, nuts, and red wine. These compounds exert health benefits primarily due to their antioxidant and anti-inflammatory properties [39,40]. Experimental and clinical studies on the effects of a polyphenol-rich diet have shown significant modulatory effects on the pathophysiological mechanisms involved in chronic diseases, such as diabetes and cardiovascular and neurodegenerative diseases, suggesting a significant prophylactic and therapeutic potential [41,42]. Given the increase in pro-inflammatory markers and reactive oxygen species (ROS) in DR, dietary intervention or supplementation with polyphenols may be a promising strategy to manage the pathology.

This review aims to comprehensively describe and clarify the effects of dietary polyphenols on the pathophysiological mechanisms involved in DR, especially those related to oxidative and inflammatory mechanisms, as well as to discuss evidence from animal models on the effects of dietary polyphenols as a prophylactic and therapeutic strategy in DR management.

## 2. Retina

The retina transduces and preprocesses the information via an intraretinal circuit and sends it via the optic nerve to the visual nuclei of the brain [43]. The mature vertebrate retina is organized into layers [44,45], such as (i) the outer nuclear layer (ONL), composed of the cell bodies of rod and cone photoreceptors, located closest to the retinal pigmented epithelium (RPE); (ii) the inner nuclear layer (INL), composed of the cell bodies of interneurons-horizontal, bipolar, amacrine, and displaced ganglion cells-located between the ONL; and (iii) the ganglion cell layer (GCL). The GCL is located near the vitreous body of the retina and consists of ganglion cells, displaced amacrine cells, and some light-sensitive ganglion cells. Interspersed between these nuclear layers are the outer plexiform layer (OPL) and the inner plexiform layer (IPL), where synaptic contacts occur. Ganglion cells are the projection neurons, and their axons form the nerve fiber layer (NFL) within the retina and give rise to the optic nerve as they exit the retina [44,46].

Photoreceptors communicate with the rod- or cone-specific bipolar cells and horizontal cells in the OPL. Bipolar cells synapse with ganglion cells in the IPL. This circuit is named the vertical/radial pathway, and all cells use glutamate as a neurotransmitter [44,47]. Horizontal and amacrine cells may modulate the radial pathway in the OPL and IPL and mediate the processing of light/image stimuli, contrast, and detection of movement direction [48].

The retinal vasculature consists of the choroidal and intraretinal blood vessels responsible for the blood supply of the outer and inner retina, respectively. In humans, the inner retina presents a peripapillary plexus in the innermost part of the NFL, accompanied by inner (in the GCL) and outer (in the IPL, INL to OPL) intraretinal beds. Interestingly, the specialized region for high visual acuity in primates, the fovea, is avascular, which minimizes light distortion [49].

**Figure 1:**
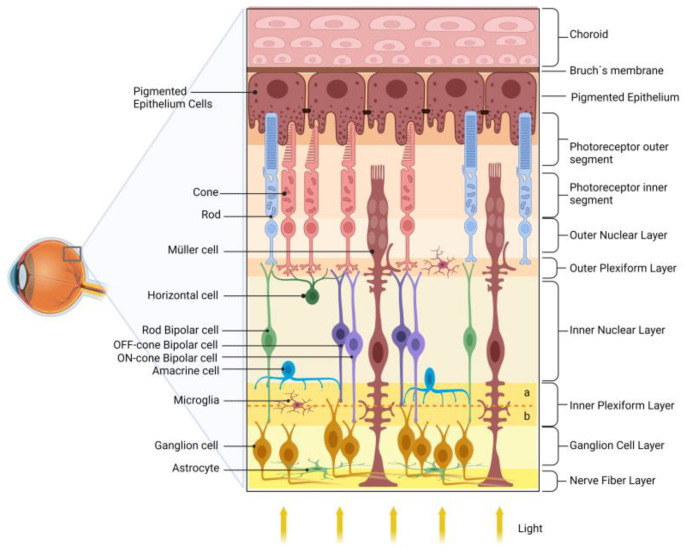
Schematic diagram of the retina. The retina is organized into nuclear layers separated by plexiform layers. The photoreceptors (rods and cones) have nuclei in the outer nuclear layer and synapse with bipolar and horizontal cells in the outer plexiform layer. Bipolar cells are divided into rod bipolar (light green) and cone bipolar (light and dark purple). Cone bipolar cells form synapses directly with their respective ganglion cells in specific sublamina (a and b, for, respectively, OFF and ON pathways) of the inner plexiform layer (IPL). Rod bipolar indirectly transmits information to ganglion cells indirectly via an amacrine cell in the IPL. The ganglion cell layer and the nerve fiber layer are formed by the nuclei and axons of ganglion cells, respectively, which transmit the information to the visual encephalic centers. The retinal glial cells are Müller cells, astrocytes, and microglia. The retinal pigmented epithelium (RPE), composed of pigmented epithelial cells, plays several essential roles in maintaining the retina’s health.

In addition to neurons, the retina contains three types of glial cells, Müller cells, astrocytes, and microglia. Müller glia, which comprises 90% of retinal glial cells, have a cell body in the center of the INL and radial processes that extend through all retina layers. Consistent with the multiple roles that Müller glia play in retinal homeostasis and physiology, dysfunction of these cells in DR may be related to retinal dysfunction and severity of DR [47,48,50,51]. Figure 1 shows a schematic drawing of the retina.

## 3. Diabetic Retinopathy

DR is a multifactorial pathology that results from a dysfunction in a variety of retinal cells, including Müller glia, ganglion cells, endothelial cells, and photoreceptors, as well as optic nerve damage and, later, retinal neuronal cell death and vascular lesions [52,53]. Although neural retina/optic nerve changes occur before vascular alterations in both human and animal models, DR is a chronic microvascular disease characterized by vasculopathy, hyperpermeability, hypoperfusion, and neoangiogenesis of slow progression, accompanied by vitreous hemorrhage, retinal detachment, diabetic macular edema process (DME), and/or macular ischemia, resulting in neural abnormalities and retinal deficits [20,54,55]. Although DME and macular ischemia can occur at any stage of the disease, they are more common in the later stages of DR [54].

Chronic hyperglycemia promotes the thickening of the retinal capillary basement membrane, impairs adherence of tight junctions between endothelial cells, and makes blood vessels more permeable. These events define the process known as the blood–retinal barrier (BRB) breakdown. This phenomenon is accompanied by decreased pericytes and mesenchymal cells surrounding endothelial cells, providing structural support for the vessel wall and vascular repair processes [56,57]. The loss of pericytes leads to voids in the capillary walls. In response, capillary endothelial cell growth is induced toward the inner part of the vessel wall, favoring the formation of cell clusters that obstruct the vessel lumen and the appearance of hemorrhagic spots and microaneurysms. This combination of damage compromises vascular function and the oxygen and nutrient supply to the retinal tissue, leading to ischemia, increased synthesis, and release of pro-angiogenic factors and neovascularization [55,57].

DR is classified based on pathophysiological characteristics and vascular manifestations during the clinical course, the initial phase, known as non-proliferative DR (NPDR), and the advanced phase, known as proliferative DR (PDR). The presence of new blood vessels characterizes the PDR stage [58].

NPDR is characterized by morphological changes and vascular damage, including alterations in the microvasculature and an increase in vascular caliber. During this phase, hyperglycemia causes hemodynamic dysfunction, increased permeability, microaneurysms, hemorrhages, and exudates. These manifestations generally progress slowly, and some patients may be asymptomatic. As the disease progresses, the frequency of hemorrhagic events increases, disrupting the retina’s oxygen supply and metabolic demands and increasing the risk of ischemic events. Fundus examination may reveal whitish spots known as “cotton-wool spots” that indicate ischemic processes or retinal infarction [56,59,60]. Thus, the abnormal angiogenic process leads to a more advanced and critical PDR [55,61].

Approximately half of NPDR patients progress to PDR in less than one year [62]. The main feature of PDR is the appearance of new blood vessels because of the local accumulation of vascular growth factors caused by prolonged hypoxia, followed by the formation of ischemic zones. These vessels are delicate, composed entirely of the endothelium, and grow along the retina and into the vitreous without initially causing symptoms or vision loss. However, because their walls are thin and fragile, the vessels can rupture and release blood into the vitreous cavity, causing severe vision loss and even blindness [55,63]. Neovascularization is usually accompanied by fibrous tissue growth, which is visible on ophthalmoscopy [62].

Although DR has been predominantly considered a microvascular disease, increasing evidence suggests that retinal dysfunction and degeneration are critical features associated with disease progression and that early neural injury occurs before vascular damage in DR [64]. Data from electroretinograms of diabetic patients show a decrease in the oscillatory potential, which would be the first observable change in the retina of these individuals [65]. Other parameters are altered in individuals with DM without vascular changes, such as contrast sensitivity, color vision, and oscillatory potentials [53]. Histologic analysis of clinical and postmortem specimens confirmed alterations in retinal structural parameters, such as changes in the thickness of different retinal layers, neuronal death, and increased oxidative stress before vascular changes visible with fundoscopy, supporting the concept of an early neurodegenerative phase of DR preceding NPDR [65,66,67,68].

In this sense, a prospective and longitudinal human study of 45 patients with type 1 DM, without or with minimal DR, reported significant and progressive impairment of both the nerve fiber layer (NFL) and the ganglion cell (GC)/inner plexiform layer, independent of glycated hemoglobin, age, and sex [64]. To confirm these findings, eye samples from six deceased patients with DM, without or with minimal DR, were compared to eyes from donors of a similar age without DM. The first group showed a significantly thinner NFL but no difference in GC density. Surprisingly, the data showed that despite the neuronal changes observed in the DR group, there was no difference in retinal capillary density between the groups. These findings were confirmed in two experimental diabetes mouse models. Recently, other studies using optical coherence tomography showed decreased retinal thickness (INL/GCL and NFL) in diabetic patients without signs of DR, indicating neurodegeneration before vascular changes [64,69,70,71,72]. These results suggest that retinal diabetic neuropathy is not necessarily a consequence of retinal ischemia. It develops gradually, independent of glycated hemoglobin, age, and sex, before the onset of microvasculopathy.

All retinal cells (e.g., ganglion, bipolar, amacrine, and photoreceptor cells) show altered function before visible microvascular lesions [53]. Therefore, the description of DR as a mere “microvascular pathology” is an oversimplified and flawed characterization that hinders the exploration of novel therapeutic approaches based, for example, on intervention on neurodegenerative pathways [73].

## 4. Cellular and Molecular Pathways Involved with Oxidative Stress and Inflammatory Response during DR

The retina is susceptible to oxidative stress due to its high polyunsaturated fatty acids (PUFA) concentration and rapid oxygen and glucose uptake. Another critical element in the pathophysiology of DR is inflammation. The oxidative stress induces ROS production, the root cause of neuropathy and retinopathy [74].

Glucose is the primary precursor for energy production in the cell. The citric acid cycle requires glycolysis-derived pyruvate to produce the high-energy electron carriers FADH_2_ and NADH, which fuel the electron transport required for ATP production. At the end of this electron transport chain, oxygen molecules serve as electron acceptors, limiting reactions that would otherwise impair the function of other cellular components. High glucose levels cause increased glycolysis rate, pyruvate levels, and FADH_2_ and NADH production. When these electron carriers reach the upper limit of the voltage gradient across the inner mitochondrial membrane, electron transfer through complex III is impaired, pushing electrons back to coenzyme Q, which mediates superoxide (O_2_^−^) production [75]. Superoxide belongs to a class of reactive oxygen species (ROS). ROS are highly reactive, mainly due to unpaired electrons in the outer shell of their atoms, causing dysfunction in enzymes and altering the interaction properties of proteins, lipids, sugars, and nucleic acids. O_2_^−^ can be converted by superoxide dismutase activity to the non-radical ROS, hydrogen peroxide (H_2_O_2_). H_2_O_2_ alters protein interactions or function through cysteine oxidation. H_2_O_2_ can be converted to hydroxyl radical (OH^.^) by the Fenton reaction, as well as to hypochlorous acid (HOCl) when targeted by neutrophil myeloperoxidase during an inflammatory process [76,77]. Indeed, diabetes-induced neuroinflammation showed increased NO synthase (NOS2) levels in the retina. The reaction of nitric oxide (NO) with O_2_^−^ forms the family member of the reactive nitrogen species peroxynitrite (ONOO−). ROS damages DNA leading to the activation of DNA repair enzymes, such as poly (adenosine diphosphate (ADP)-ribose) polymerase. This enzyme produces ADP-ribose, which inhibits glycerol-3-phosphate dehydrogenase (GAPDH), which is responsible for a step in the glycolytic pathway. The result is an accumulation of glycolytic intermediates used by the polyol and hexosamine pathways, advanced glycation end products (AGEs), and upregulation of the PKC signaling pathway [78]. 

All these pathways increase ROS and form a positive feedback loop to increase oxidative stress in the cell. Among many ROS-generating systems, NADPH oxidase (NOX) activity seems particularly important for generating cytoplasmic ROS, especially for neovascularization [79]. NOX is a group of enzymes that catalyzes the production of O_2_^−^ by transferring an electron from NADPH to O_2_. NOX4, a subtype of NOX, can also produce H_2_O_2_. NOX activity is upregulated by several signals produced during the progression of DR, including tumor necrosis factor-alpha (TNF-α), several interleukins, and AGE signaling. Therefore, NOX appears to be important in advance of DR, and inhibition of NOX has emerged as a promising therapeutic target for DR [80,81]. Thus, oxidative stress is a constitutive problem and a hallmark of DM. 

As mentioned above, hyperglycemic conditions induce the production of AGEs. Briefly, AGEs are generated by the reduction in amino acids, lipids, or nucleic acids by glucose, triggering the formation of Schiff bases. Rearrangements of Schiff bases lead to forming Amadori products, which have reactive carbonyl groups. These carbonyl groups interact with amino, sulfhydryl, and guanidine functional groups, denaturing and cross-linking target proteins. Finally, reactions of Amadori products with arginine or lysine within target proteins lead to the formation of stable AGEs. For example, AGE can signal through specific receptors, the receptor for advanced glycation end products (RAGE), mediating paracrine and autocrine signaling involved in the pathology of diabetic retinopathy [82].

AGEs and oxidative stress activate retinal micro- and macroglia during diabetic retinopathy [83]. Once activated, microglia produced interleukin (IL)-1β, IL-6, IL-8, and TNFα under the control of NF-kappaB (NF-kB) [84,85,86,87,88,89]. Müller glia is activated in DR, leading to increased secretion of IL-6, IL-8, VEGF, INF-γ, TNF-α, IL-1β, monocyte chemoattractant protein-1 (MCP-1), and EGF [90,91]. In addition, galectin-3 is upregulated in Müller glia in diabetic rats, and the absence of galectin-3 reduces the number of astrocytes and macrophage-like cells, both quiescent and activated, in the optic nerve and protects against ganglion cell death [92]. Retinal astrocytes are consistently activated by oxidative damage, increasing their secretion of IL-6, MCP-1, and MIP-2 under the control of p38MAPK and NF-kB signaling [93]. In addition, the AGE receptor mediates astrocyte activation biased toward a pro-inflammatory profile, increasing NF-kB and complement C3 expression, mediating synaptic loss [94].

All these secreted mediators orchestrate various pathological events leading to persistent inflammation, ROS production, neurodegeneration, blood–retinal barrier permeability, and defective neovascularization, which are hallmarks of diabetic retinopathy. TNF-α and galectin-3 mediate apoptotic cell death of retinal ganglion cells [92,95]. Both IL-1β and TNF-α increased the content of ICAM-1 on the endothelial surface, allowing monocyte diapedesis through VLA4 and CD18 binding [96]. These monocytes are attracted to the retina by MCP-1 secreted by astrocytes and Müller glia and differentiate into macrophages once in the retinal tissue [97]. IL-8 recruits neutrophils, which release myeloperoxidase granules, further contributing to the maintenance of oxidative stress [98,99]. These leukocyte recruitments rely on the luminal exposure of MIP-2, which stimulates the upregulation of leukocyte integrins during diapedesis [100]. Oxidative damage and IL-6 stimulate VEGF production by Müller glia and endothelial cells, respectively [101]. VEGF decreases occludin content in endothelial cells, thereby increasing the permeability of the blood–retinal barrier, leading to retinal edema and degeneration [102]. To worsen the scenario, VEGF and EGF stimulate neovascularization, expanding the area covered by defective capillaries in the proliferative phase of diabetic retinopathy [103]. Thus, neuroinflammation and oxidative stress produce each other in a positive feedback loop, contributing to neuronal death and vascular pathology of diabetic retinopathy [53].

## 5. Dietary Polyphenols: A Potential Strategy for Management of Diabetic Retinopathy?

**Figure 2:**
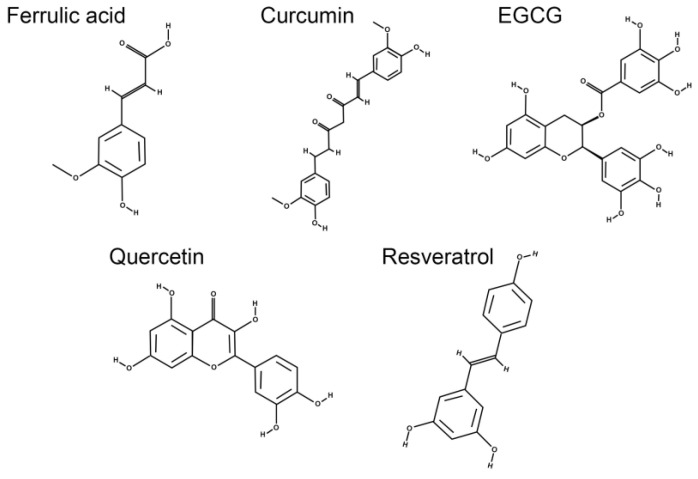
Chemical structures of polyphenolic compounds with potential therapeutic implications in diabetic retinopathy: ferulic acid, quercetin, resveratrol, curcumin, and epigallocatechin gallate.

Early studies have demonstrated the importance of consuming foods that contain substances with antioxidant properties for the prevention of cardiovascular disease, cancer, and age-related degenerative brain disorders [104,105,106]. Natural products and plant extracts with antioxidant properties, low toxicity, and known health-promoting properties have been suggested as possible treatments for eye diseases [107,108,109,110,111,112,113]. Although polyphenols offer several advantages, it is crucial to consider certain factors when evaluating their physiological effects. Some polyphenols have low water solubility and limited oral bioavailability, requiring higher doses, which are significant barriers to their broader use in therapies [114,115]. Furthermore, their physicochemical stability, gastrointestinal absorption, and metabolism are crucial to ensure their efficacy [116,117]. In this context, given the wide range of biological activities associated with these molecules (Figure 2), it seems plausible to study not only the potential health benefits but also to develop strategies and tools (pharmacotechnical and biotechnological) to overcome the limitations mentioned above, accompanied by research on the molecular mechanisms of dietary polyphenols and/or their supplementation in diseases affecting the human eye, such as DR.

### 5.1. Ferulic Acid

Ferulic acid (FA) was first isolated from Ferula foetid in 1866. FA is a polyphenol of the hydroxycinnamic acid (HCA) family, derived from secondary metabolites of lignin biosynthesis from phenylalanine and tyrosine, found in the free or conjugated form [118,119,120]. FA has shown various health biological and therapeutic properties, including anti-inflammatory, antioxidant, antibacterial, anti-diabetic, anti-carcinogenic, anti-aging, and neuroprotective [121].

FA is a potent antioxidant by neutralizing free radicals and enhancing the cellular stress response by upregulating cytoprotective systems, such as heme oxygenase-1, heat shock protein 70, extracellular signal-regulated kinase ½, and the proto-oncogene Akt. In addition, FA can suppress the expression and/or activity of cytotoxic enzymes, such as cyclooxygenase-2 (COX-2), caspases, and inducible nitric oxide synthase. (iNOS) [122]. In animal models of hepatocytic and aortic inflammation and ulcerative colitis, FA treatment inhibited the production of the inflammatory cytokines IL-6, IL-1, and TNF-α. The ability of hydroxycinnamic acids, including FA, to inhibit the NF-kB pathway and suppress the expression of COX-2 and iNOS may be a representative anti-inflammatory mechanism [123]. Using optical coherence tomography and electroretinography, FA or its analog ethyl ferulate attenuated morphologic and functional retinal degeneration in mice, as evidenced by reductions in outer retinal and choroidal layer thickness [124].

Retinal degeneration in DR is typically accompanied by glia-mediated neuroinflammation. FA supplementation reduces neuroinflammation by suppressing microglial activation in mice [125]. The effect on human retinal pigment epithelial cells has also been studied. A recent study demonstrated that FA exerts a potent protective effect against oxidative stress-induced retinal damage. The authors showed that preincubation of human retinal pigment epithelial (ARPE-19) cells in culture with FA for 24 h significantly attenuated apoptosis induced by four h of H_2_O_2_ challenge by upregulating Bcl-2 and downregulating Bax [126].

Zhu et al. [127] investigated the anti-apoptotic effect of FA in RPE cells (ARPE-19) exposed to high glucose and its cytoprotective effects in the retina of db/db diabetic mice. Treatment with FA significantly increased cell viability and suppressed cell apoptosis in ARPE-19 cells compared with the untreated group. In addition, treatment with FA (0.05 g/kg for two months) alleviated degenerative changes in the retinal tissues of db/db mice. It sharply decreased the expression of apoptosis-related markers in the retinal tissues compared to the control group. The effect shown in the FA-treated groups was related to P53 and BAX inactivation and Bcl2 activation.

Adenosine monophosphate-activated protein kinase (AMPK) is a potent regulator of metabolism and an attractive target for antidiabetic drugs. Hyperglycemia induces oxidative stress and decreases AMPK phosphorylation. Sigh and Patil [128] showed that trans-FA increased cellular phosphorylation of AMPK in cultured HepG2 and L6 cells exposed to high glucose in a time-dependent manner, besides causing upregulation of GLUT2 and GLUT4 and inhibition of JNK1/2 activity.

In diabetic patients, the formation and accumulation of AGEs are related to the onset and development of atherosclerosis by inducing oxidative damage, vascular contraction, and blood coagulation state, stimulating the release of cytokines, and increasing the inflammatory process in blood vessels. Reducing or inhibiting AGE formation may be a prospective strategy to prevent or delay the development of vascular complications in DR.

Liu et al. [129] have demonstrated an inhibitory effect of FA on AGE formation in vitro. Pretreatment with FA prevented AGE-induced p38 MAPK signaling and NF-κB pathway activation in human vascular endothelial cells (HUVEC). Because of the many possibilities, researchers have worked on strategies for the ocular delivery of FA by microencapsulation. This drug delivery system eliminates losses during the digestive process and increases in situ bioavailability [130]. Table 1 summarizes the findings for the use of FA in DR.

### 5.2. Curcumin

Curcumin (1,7-bis[4-hydroxy-3-methoxyphenyl]-1,6-heptadiene-3,5-dione), also known as diferuloylmethane, Natural Yellow 3, or E100, according to the European coding of food additives, belongs to the group of polyphenols. It was first isolated from the rhizome of *Curcuma longa* L. (turmeric) in 1815, the main component of this plant species. It is mainly grown in India and China, and it was first known for its role as a food additive or spice in cooking (e.g., curry) [131,132]. Clinical studies have reported that curcumin exerts anticancer, anti-inflammatory, and antioxidant effects. Furthermore, curcumin has a potential neuroprotective effect [133]. Studies in diabetic mouse models have shown that oral administration of various doses of curcumin reduces blood glucose levels [134] and HbA1C levels [135] and improve insulin sensitivity [136,137]. Dietary curcumin also effectively reduced fasting blood glucose, urine glucose, and urine volume in STZ-induced diabetic rats [138].

Early evidence has shown that using curcumin as a therapeutic agent can help to improve the condition of patients with retinal diseases [139,140,141,142]. Many of these effects have been demonstrated using RPE cells and the retina of diabetic rats as models. Curcumin may exert its pharmacological properties by inhibiting cell proliferation and angiogenesis by targeting molecular and cellular pathways involved in the pathogenesis of retinal diseases [143].

Under hypoxic conditions, HIF-1 plays a significant role in the expression of VEGF [144,145,146], and studies have described how curcumin can decrease VEGF production through the control of HIF-1 [147,148]. The progression of DM is associated with increased ROS levels in the neural retina and vascular endothelium [149,150]. These events may decrease the antioxidant defense system and enhance oxidative damage in diabetic retinopathy [143,151,152].

Curcumin has a potent anti-inflammatory effect by downregulating the expression of TNF-α, IL-1, IL-6, and IL-8. In addition, curcumin can downregulate the cellular action of angiogenic proteins, such as VEGF, by inhibiting transcription factors, including NF-κB, HIF-1α, AP-1, and early growth response-1 (EGR-1) [153,154,155]. Curcumin treatment reduced NFκ-B and phosphorylated NFκ-B, IKB-α, IL-1β, and Bax levels, while increasing Bcl-2 expression in rat retinal vascular endothelial cells (RRVECs) exposed to high glucose [156].

In addition, Ran et al. [157] observed that curcumin downregulated high glucose-induced inflammatory injury in ARPE-19 by modulating the ROS/PI3K/AKT/mTOR signaling pathway and decreasing the levels of pro-inflammatory cytokines TNF-α, IL-1β, and IL-6. These findings show that curcumin exerts anti-inflammatory effects by modulating multiple signaling pathways, making it a promising molecule against inflammation and oxidative stress in DR.

Under physiological conditions, the nuclear erythroid 2 p45-related factor (Nrf2) is an essential protein located primarily in the cytoplasm. In response to oxidative stress, Nrf2 primarily modulates endogenous defense. Nrf2 translocates to the nucleus and binds to specific DNA sites, such as electrophile response elements (EpRE) or antioxidant response elements (ARE), to initiate the transcription of cytoprotective genes [158,159]. Then, 1,5-bis(2-trifluoromethylphenyl)-1,4-pentadien-3-one(C3), a curcumin analog, inhibited the cytotoxic effects of acrolein in the ARPE-19 cells by directly activating the Nrf2 signaling cascade. More effectively than curcumin, it increased GSH and mitochondrial reductive enzymes while protecting ARPE-19 cells from oxidative stress [160].

Curcumin may help treat DR since it increases Nrf2/HO-1 and ERK1/2 expression and reduces ROS-induced apoptosis in RPE cells [161]. Xie et al. [162] investigated the effect of curcumin on retinal damage in streptozotocin (STZ)-induced diabetic rats and found that four weeks of oral curcumin, subcutaneous insulin, or combination therapy improved histopathologic parameters, oxidative stress markers, and reduced plasma glucose in all groups studied. In addition, curcumin had a better anti-photoreceptor apoptosis effect compared to insulin treatment. Curcumin has a regulatory impact on oxidative stress-induced activation of the Nfr2 pathway and inhibition of the AGE–RAGE pathway and extracellular matrix (ECM)-receptor interaction in the diabetic retina. Table 2 summarizes the findings for the use of curcumin in DR.

### 5.3. Flavonoids

Polyphenols are classified into flavonoids (e.g., catechins, isoflavones, apigenin, quercetin, lutein, and anthocyanins) and non-flavonoids (e.g., chlorogenic acids in coffee) [163]. Flavonoids represent one of the main groups of antioxidants found in plants and are divided into seven subclasses, flavonols, flavones, isoflavones, anthocyanidins, flavanones, flavanols, and chalcones (these molecules are precursors of flavonoids and isoflavonoids) [164]. In addition to antioxidant activity, flavonoids have important biological activities, such as anti-inflammatory, antimicrobial, anticarcinogenic, and immunostimulatory effects [165]. The consumption of flavonoids is inversely associated with cancer, cardiovascular disease, and neurodegenerative disorders [166].

#### 5.3.1. Epigallocatechin Gallate

Flavanols (flavan-3-ols) differ from other flavonoids by having a hydroxyl in position 3 of the C ring and no double bond between positions 2 and 3 of the same ring. These compounds are found as single molecules (glycated or aglycone) or organized in polymers. Among the flavanols, the catechin groups (catechin, catechin gallate, epicatechin, epicatechin gallate, epigallocatechin, and epigallocatechin gallate), proanthocyanidins, theaflavins, and thearubigins stand out. Food sources of flavanols include tea (*Camellia sinensis*), grape seeds, cherries, berries, and the skins of various fruits [164].

Epidemiological studies associated *C. sinensis* consumption, mainly green tea, with a reduced risk of several types of cancer [167,168], and its polyphenols are effective chemoprevention agents [169,170]. Other properties include antiallergic, antimicrobial, immunostimulatory, and anti-inflammatory effects [171], as well as increased insulin activity [172] and protection against cardiovascular and brain disease [173,174].

Retinal GSH levels and activity of the antioxidant enzymes SOD and CAT are reduced in diabetic rats. The administration of green tea extract at 200 mg/kg daily for 16 weeks to diabetic rats restored GSH levels. It almost increased the SOD and CAT enzyme activities to the same levels as the non-diabetic group. In addition, green tea treatment led to a reduction in the expression of pro-inflammatory markers TNF and VEGF in the retina and a decrease in the thickness of the retinal capillary basement membrane [175].

According to Silva et al. [176], daily consumption of green tea (5.7 g/kg) for 12 weeks reduced retinal oxidative stress, prevented the upregulation of GFAP and the downregulation of occludin and restored the expression of activated neuronal nitric oxide synthase (nNOS) in diabetic mice. Green tea regulated the AKT-dependent activation of the endothelial enzyme nitric oxide synthase (eNOS). NO produced by eNOS activation and inflammatory markers are required for VEGF-induced increased vascular permeability, which contributes significantly to BRB breakdown. In addition, green tea restored glutamate transporter, glutamate receptor, and glutamine synthetase.

Many of the beneficial effects promoted by the consumption of green tea may be due to several antioxidant compounds in its composition, most notably EGCG, as evidenced by in vitro studies that evaluated the effect of incubating retinal cells with EGCG in a hyperglycemic medium. Chan et al. [177] observed that incubation of RPE with 10 µM EGCG inhibited the migration of adult ARPE19 cells and decreased the adhesion of epithelial cells to fibronectin. In addition, EGCG (3 µM) effectively inhibited the migration of RPE cells by preventing the activation of PI3K/Akt, ERK1/2, and p38 phosphorylation and blocking the MAPKs pathway.

Wang et al. [178] demonstrated that EGCG at 10 µM promoted autophagy in rat retinal Müller cells incubated in a hyperglycemic medium. The authors found that EGCG treatment decreased P62 protein levels, maintained LC3-B and Beclin-1 protein levels, and stimulated autophagosome and autolysosome formation. The EGCG-induced decrease in the mTOR pathway can explain these effects on autophagy.

#### 5.3.2. Quercetin

Compared to other flavonoids, flavonols (3-hydroxyflavones) have a higher concentration of hydroxyls in the A and B rings. Quercetin, myricetin, kaempferol, and galanin are some of the primary representatives of this class, which are present in different foods, such as tea (*Carmellia sinensis*), onions, apples, and berries [179,180].

Quercetin can exert several biological effects, including anti-inflammatory, antioxidant, antidiabetic, anti-hypertensive, anti-obesity, anti-hypercholesterolemic, anti-atherosclerotic, anti-cancer, and anti-tumor [181,182,183]. In addition, quercetin consumption has been associated with protection against retinal ischemia-reperfusion injury, inflammation, and age-related ophthalmopathy [184,185].

Cheng et al. [186] showed that ARPE-19 cells incubated with quercetin (2.5–20 µM) could dose-dependently reduce IL-1β-induced expression of inflammatory markers, such as ICAM-1, sICAM-1, IL-6, IL-8, and MCP-1. This phenomenon was associated with decreased phosphorylation of MAPKs (ERK1/2, p38, and JNK1/2) and cAMP response element-binding protein (CREB).

Chai et al. [187] found that intraperitoneal administration of quercetin (150 mg/kg) for 16 weeks reduced the number of apoptotic cells and improved the thickness of both the inner and outer nuclear layers. Such retinal histopathologic changes were accompanied by a decrease in the pro-inflammatory cytokines IL-1, IL-18, IL-6, and TNF-α, as well as in the production of the pro-angiogenic factors and soluble adhesion proteins VEGF and sICAM-1. In addition, quercetin treatment increased the expression of neurotrophic factors, including brain-derived neurotrophic factor (BDNF) and nerve growth factor (NGF), suggesting that quercetin may have a neuroprotective effect in DR. The results also showed that quercetin supplementation increased the expression of heme oxygenase-1 (HO-1), decreased the expression of both HMGB-1 and TLR4, and reduced the hyperactivation of NLRP3 and NF-kB inflammasomes. The activation of HO-1 by quercetin may contribute to some of its effects. Treatment with zinc protoporphyrin (ZnPP), an HO-1 inhibitor, prevented the retinal protective effects of quercetin. The results suggest that quercetin may have therapeutic effects in DR by increasing the expression of HO-1.

The formation of retinal neovascularization (RNV) is a hallmark of PDR. In this context, understanding the mechanisms involved in retinal angiopathy and inflammation associated with DR has become essential in developing therapeutic strategies. High glucose conditions promote the activation of angiogenic mechanisms and increase the expression of NLRP3, ASC, caspase-1, IL-1, and IL-18, as well as the autophagic proteins LC3-B and Beclin-1. Quercetin treatment effectively inhibits the activation of angiogenic, inflammatory, and autophagic mechanisms induced by hyperglycemia in a dose-dependent manner (20 to 80 µmol/L) [188]. The results may suggest that the therapeutic potential of quercetin for retinal neovascularization in DR involves the inhibition of NLRP3 inflammation and autophagy signaling.

Chen et al. [189] demonstrated that STZ-induced diabetic rats receiving quercetin supplementation had decreased expression of matrix metalloproteinase-9 (MMP-9) and VEGF proteins. However, quercetin did not affect the expression of the inflammatory mediator MCP-1.

Daily five-week quercetin supplementation (50 mg/kg) increased retinal BDNF, NGF, and tropomyosin-related kinase B (TrkB) in diabetic rats. The treatment also prevented apoptosis by reducing cytochrome c and caspase-3 activity while increasing levels of the anti-apoptotic protein Bcl-2. In addition, quercetin treatment significantly increased retinal GSH levels compared to untreated diabetic rats. The results suggest that quercetin may protect neurons in the diabetic retina by increasing neurotrophic factors and preventing neuronal death through potential anti-apoptotic effects mediated by the BDNF-TrkB/Akt-synaptophysin pathway [190].

Like EGCG, quercetin inhibits photoreceptor cell apoptosis by inhibiting the PI3K/Akt pathway. By stimulating Bcl-2, which prevents Bax-mediated apoptosis, quercetin most likely suppresses the PI3K/Akt signaling pathway [191]. Another pathway of quercetin is the activation of sirtuins, a protein involved in DNA repair and regulation of metabolic processes. Sirtuin 1 protein is found in the outer nuclear layer and is secreted in the early stages of retinal degeneration, aiding the repair process [192]. Table 3 summarizes the findings for the use of quercetin in DR.

### 5.4. Resveratrol

Resveratrol (3,5,4 trihydroxystilbene) is a non-flavonoid polyphenol found in grapes, peanuts, blackberries, and blueberries. Its high concentration in red wine has been associated with a lower risk of cardiovascular disease in European populations who consume the beverage frequently [193,194]. Resveratrol consists of two aromatic rings connected by a methylene bridge. The molecule naturally has two isomers, cis and trans-resveratrol, the latter being the biologically active form [195,196].

Early studies have reported several beneficial health properties of resveratrol, including anti-tumor, anti-aging, anti-inflammatory, antioxidant, cardioprotective, and neuroprotective [195,197,198]. In addition, resveratrol has been shown to modulate angiogenesis, proliferation, and cell death [194].

Streptozotocin-induced diabetic rats have increased blood glucose, glycosylated hemoglobin, fructosamine, advanced glycation end products (AGEs), and decreased body weight. At different time points (1–7 months), these parameters were partially inhibited by the administration of resveratrol diluted in saline (5, 10, and 20 mg/kg per day) [199,200,201,202,203] or coated with gold nanoparticles (300 mg/kg per day) [204]. Furthermore, after months of diabetes, the retina of these animals showed intense cell death, concentrated in the INL and the GCL, as demonstrated by various techniques [199,204]. The death induced by high glucose is probably due to increased production of pro-inflammatory cytokines (TNF-α, VEGF, INFγ, MCP-1, IL-6, IL1-β, and COX-2) and increased expression and activity of NF-kB [200,201,203,204].

An imbalance between ROS production and antioxidant defenses has been reported in the blood and retina of diabetic rats, with a decrease in SOD activity and an increase in the ratio of GSSH/GSH and 8-isoprostane levels, an indicator of oxidative stress [199,200,204]. Hyperglycemia also contributed to the rise in vessels in the ganglion cell layer, probably due to the increase in VEGF [204]. Resveratrol has been shown to reverse functional, morphological, and molecular effects in the retina of diabetic rats [199,200,201,202,204]. It appears that inhibition of NF-kB activity contributes to many of the neuroprotective effects of resveratrol in diabetes by reducing the levels of proteins associated with inflammation and cell death [200,201,204].

Data support the anti-inflammatory action of resveratrol and suggest that this is an essential mechanism for preventing high glucose-induced cell death. On the other hand, the neuroprotective activity of resveratrol may reflect a joint effort of different effects. In this sense, it has already been shown that resveratrol inhibits apoptosis in the retina of diabetic rats and Müller cell cultures by increasing microRNA 29b, which decreases pro-apoptotic proteins (SP1, activated caspase-3, and Bax) and increases anti-apoptotic (Bcl-2) proteins [205].

In vitro studies with endothelial cell lines showed that the protective effect of resveratrol also depends on the activation of sirtuins (SIRT), a classical target of this polyphenol. Bovine retinal capillary endothelial cells (BRECs) exposed to high glucose (30 mM) died by apoptosis induced by increased production of ROS. High glucose also reduces the expression of p-AMPK, SIRT-1, and PGC-1α, leading to cell death [206]. It has been demonstrated that the treatment with resveratrol (20 µM) prevented the reduction in protein levels of p-AMPK, SIRT-1, and PGC-1α, and blocked ROS-induced cell death [206]. A decrease in SIRT-1 levels was also observed in peripheral blood mononuclear cell cultures from patients with proliferative diabetic retinopathy and an increase in interleukin-17, a pro-inflammatory cytokine. The effects were reversed when these cells were exposed to resveratrol (10 µM) [207]. In human retinal microvascular endothelial cells (HRECs), exposure to high glucose (25 mM and 30 mM) resulted in decreased mRNA expression for mitochondrial SIRT-3, 4, and 5, which was accompanied by early endothelial senescence [208]. There was also oxidative damage and increased transition from endothelial to mesenchymal cells, contributing to tissue fibrosis resulting from increased glucose [208,209]. The phenotypic change from an endothelial to a mesenchymal cell depends on ROS production through NADPH oxidase activity via PKC. In this case, resveratrol treatment inhibited this phenotypic transition by inhibiting PKC activation [209]. However, whether the activation of AMPK/SIRT is also involved in this phenomenon is unknown. Therefore, more evidence is needed to answer this question.

Resveratrol has excellent therapeutic potential against abnormalities induced by high glucose in experimental models of diabetes. Its protective effects include neuronal, vascular endothelial, and pigmented epithelial cells by activating different pathways that may or may not be complementary. What would be the primary molecular target of resveratrol, although SIRT is a strong candidate? In any case, resveratrol has an anti-inflammatory effect combined with its antioxidant effect under high glucose conditions. The sum of these effects probably contributes to its overall protective effect. Table 4 summarizes the findings for the use of resveratrol in DR.

**Figure 3:**
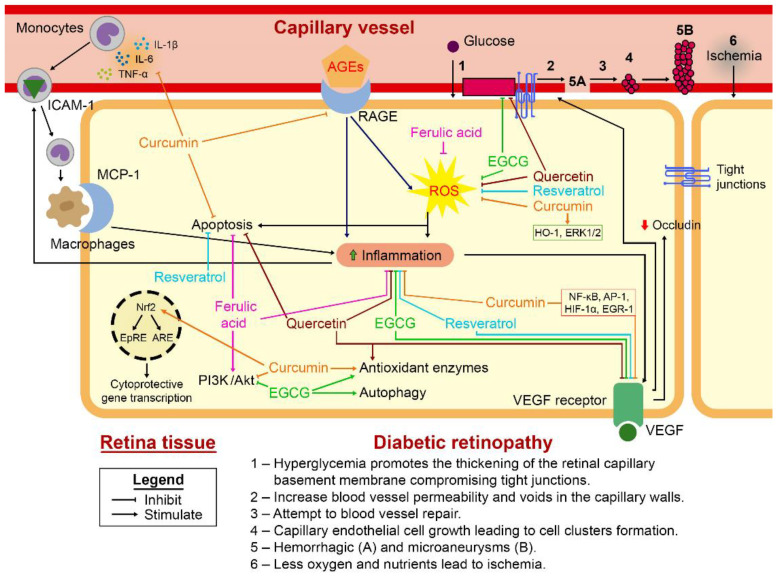
Schematic illustration of cellular and molecular pathways in diabetic retinopathy leading to oxidative stress, inflammation, and apoptosis. Polyphenols, such as quercetin, curcumin, resveratrol, ferulic acid, and epicatechin gallate, contribute to the inhibition of positive feedback and thus reduce the occurrence of cellular signaling cascades involved in diabetic retinopathy.

Lastly, Figure 3 shows a schematic diagram illustrating the cellular and molecular pathways in diabetic retinopathy leading to oxidative stress, inflammation, and apoptosis and how polyphenols act to downregulate these effects.

### 5.5. Lutein and Zeaxanthin

Lutein and zeaxanthin are carotenoids that have essential functions in eye health and are available as dietary supplements or foods [210]. In addition to potential antioxidant action, these compounds are found in the human retina and absorb wavelengths emitted by light, contributing to eye protection [211]. Individuals with type 2 DM have lower macular pigments, such as lutein and zeaxanthin, than healthy subjects [212].

Studies have demonstrated the benefits of lutein and zeaxanthin supplementation in diabetic retinopathy. Both short- and long-term supplementation with lutein and zeaxanthin in rodents reduced inflammatory biomarkers and vascular damage in the retina [213,214]. In addition, lutein and zeaxanthin supplementation reduced oxidative stress and inflammation, increased neuroprotection, decreased production of reactive oxygen species, increased GSH, peroxidase, and SOD levels in the retina of diabetic rats [215,216,217,218,219,220,221], as well as inhibited retinal histologic changes and increased retinal thickness [222]. The protective mechanisms of lutein and zeaxanthin in preventing eye diseases, such as cataracts, age-related macular degeneration, and diabetic retinopathy, have been reported [223].

## 6. Potential Interactions among Polyphenols, Probiotics, and Gut Microbiota in DR

The gastrointestinal tract is a primary site of action of polyphenols [224]. In this environment, polyphenols can interact beneficially with the gut microbiota and exert health benefits in the gut and beyond. However, the bidirectional relationship between gut microbiota and polyphenols is not straightforward. The beneficial health effects of polyphenols on the gut microbiota depend on numerous factors, such as their chemical structure, glycation pattern, and number of hydroxyl groups [224]. Similarly, gut dysbiosis, a condition characterized by an impairment of gut microbial composition and altered functional capacity that exceeds the resilience and resistance of the gut, can impair the metabolism and bioavailability of polyphenols.

Early evidence has suggested the role of gut dysbiosis in developing and maintaining DR [225,226,227]. Although previous studies have reported antioxidant and anti-inflammatory benefits with polyphenol therapy in DR, to our knowledge, there are no studies evaluating the role of microbiota in the reported effects of polyphenol therapy in DR. This gap should be explored in further clinical and animal studies.

Probiotics, prebiotics, and symbiotic consumption can alleviate intestinal dysbiosis, inflammation, and oxidative stress. In addition, selected probiotic strains can improve the metabolism of polyphenols. In contrast, polyphenols can positively modulate the composition of the gut microbiota, with effects like those of known prebiotics [228]. In addition, polyphenols can act as prebiotics and have been shown to regulate intestinal metabolism and immunity, besides influencing inflammatory pathways by altering T-cell functions, inhibiting mast cell degranulation, and reducing inflammatory cytokine responses [229,230,231,232].

In this way, the combined intake of probiotic strains and polyphenols has been considered an approach for a bi-directional improvement of their health-promoting effects [233] (Figure 2). Although an early randomized, controlled, crossover study found an attenuated early-phase insulin response after ingestion of probiotic fruit drinks with different polyphenol profiles in healthy young adults [234], whether combined therapy with pro-pre-symbiotic and polyphenols reduces oxidative stress, inflammation, and diabetic complications in DR remains to be elucidated.

**Figure 4:**
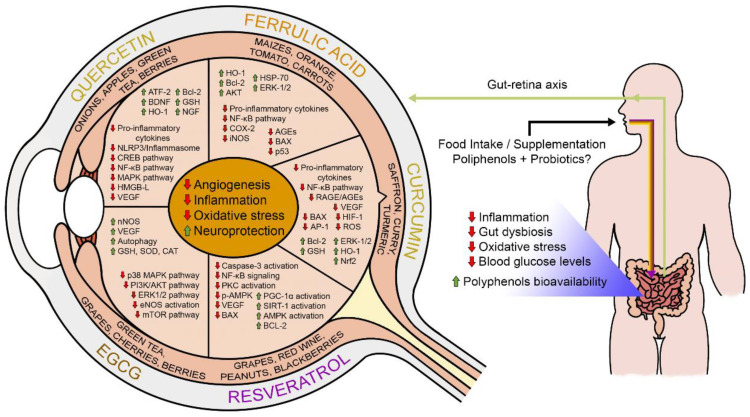
Food sources of polyphenols and the health benefits caused by the consumption of these substances are associated with probiotics, with the main results of reducing blood sugar levels, inflammation, oxidative stress, angiogenesis, intestinal dysbiosis, and increasing neuroprotective factors.

Considering the early clinical evidence that probiotics, prebiotics, symbiotics, and polyphenols may alleviate glycemic dysfunction in DM conditions [235], while preclinical and clinical studies evaluating the effects of combined probiotics and polyphenols [236,237,238] in DR are not conducted, it is reasonable to suggest that DR patients may benefit from daily consumption of probiotics and dietary polyphenols for better glycemic control (Figure 4).

## 7. Conclusions

The potential of polyphenols as an efficient, non-invasive, and inexpensive treatment approach in the face of ophthalmologic pathologies is evident based on the results from the available literature discussed in the present review. It appears that dietary and/or nutritional supplementation with polyphenols, alone or in combination with probiotics, for example, may slow the progression of retinal diseases. The beneficial effects of the antioxidants studied seem to act indirectly rather than directly in controlling diabetic retinopathy through the activation of metabolic pathways responsible for increasing the antioxidant capacity of cells. Specifically in the retina, these antioxidant compounds stimulate several retinal anti-inflammatory pathways leading to inhibition/reduction in cell death caused by inflammation, AGEs, and hyperglycemia, in addition to improving membrane permeability and retinal cell structure. In addition, understanding the molecular mechanisms and how polyphenols affect the signaling pathways involved in neuroinflammation and oxidative stress may help develop effective therapeutic options for various retinopathies, including diabetic retinopathy. The importance of performing well-defined clinical trials to evaluate the efficacy, safety, dose range, and potential side effects, as well as chemical and pharmacokinetic analyses to improve the stability and bioavailability of these compounds, is emphasized.

Polyphenols are extensively metabolized in the gastrointestinal tract and liver, resulting in low systemic levels of active compounds reaching target organs, such as the retina. Strategies to increase the bioavailability of polyphenols, such as nanoformulations or combination with absorption enhancers, appear promising for this challenge. Nevertheless, the beneficial effects of polyphenols in various models of DR are evident and suggest that, despite the complexity of their molecular structure, they can cross the blood–retinal barrier. The literature lacks information on the bioavailability and metabolism of these polyphenols in the retina, so studies to address this issue are essential. One strategy would be to use high-performance liquid chromatography, which has been used to assess the levels of other drugs in the retina, to assess polyphenol levels in animal studies and specifically in retinal tissue. Furthermore, it is possible that the product of the metabolism of these polyphenols by the cytochrome P450 system in the retinal tissue has beneficial biological effects and that the protective effect is a synergistic response of these produced compounds. Another relevant point is correlating the doses administered in animal models with the dose and human consumption from different food sources.

On the other hand, the beneficial effects of polyphenols and other drugs of natural origin on the retina are much more associated with their chronic consumption than with punctual administration. In this sense, additional preclinical and clinical research is needed to discover the ideal dosage and treatment duration for optimal results, which is challenging to establish a uniform methodology. Furthermore, due to the multifactorial nature of DR, relying on a single polyphenol may not sufficiently inhibit the various risk factors involved. Therefore, a dietary intervention using a combination of polyphenols may offer advantages in preventing sight-threatening retinal diseases. By simultaneously suppressing multiple risk variables, this approach has the potential to be more effective in mitigating the complexities associated with DR. Another issue to consider is the interaction of polyphenols with medications. DR often requires multiple medications, such as insulin, oral hypoglycemic agents, or blood pressure medications. The interaction of polyphenols with various medications must be studied to ensure their safety and efficacy. It is also important to investigate the compatibility of polyphenols and their potential synergistic or antagonistic effects with other drugs.

Finally, genetic and environmental factors may cause individuals to respond differently to polyphenol treatment. Several factors, such as gut microbiota composition, genetic polymorphisms related to polyphenol metabolism, and individual dietary patterns, may influence the therapeutic benefits of polyphenols. Individual differences may require personalized approaches or targeted interventions to optimize outcomes. This information will help clarify whether polyphenol interventions could be an adjuvant and effective strategy for improving glycemic parameters and the quality of life of people with diabetes.

## Figures and Tables

**Table 1:** Primary outcomes of the studies testing the use of ferulic acid in diabetic retinopathy.

Compound	Food Source	Effects on the Retina		
		Experimental Model	Effects	Reference
Ferulic acid	Cereals (maize, rice endosperm, barley extract), grasses, oranges, tomatoes, carrots, sugar-beet pulp, and bamboo shoot	Mice	Attenuated the NaIO_3_-induced retinal degeneration in the retina and choroid.	[124]
		Mice, intragastric administration.	Suppression of microglia activation.	[125]
		Human retinal cells.	Protection against oxidative stress-induced retinal damage. Reduction in H_2_O_2_-induced apoptosis by upregulating Bcl-2, downregulating Bax, and cleaving caspase-3 protein expression.	[126]
		RPE cells incubated in 30 mmol/L glucose medium.	Decreased cell apoptosis and lowered P53, BAX, and Bcl2 expression levels.	[127]
		db/db diabetic mice.	Decreased degenerative alterations in the retinal tissues. Decreased expression of apoptosis-related markers in retinal tissues.	
		HepG2 and L6 cells incubated in a hyperglycemic medium.	Increased phosphorylation of AMPK, upregulation of GLUT2 and GLUT4, and inhibition of JNK1/2 activity.	[128]
		HUVEC cells incubated in a hyperglycemic medium.	Inhibitory effects on AGEs and p38 MAPK signaling pathway, activating NF-κB pathway.	[129]

**Table 2:** Primary outcomes of the studies testing the use of curcumin in diabetic retinopathy.

Compound	Food Source	Effects on the Retina		
		Experimental Model	Effects	Reference
Curcumin	Rhizome of *Curcuma longa* L.	RPE cells and the retina of diabetic rats	Inhibited cell proliferation and angiogenesis by modulating cellular signaling pathways.	[143]
		RRVECs incubated in a hyperglycemic medium	Reduced levels of NFκ-B and pNFκ-B, IKB-α, IL-1β, and Bax, and increased expression of Bcl-2,	[156]
		RRVECs incubated in a hyperglycemic medium Diabetic rats	Improved basal membrane thickness.	[154,156]
		ARPE-19 cells	Downregulated ROS/PI3K/AKT/mTOR signaling pathway by decreasing the levels of pro-inflammatory cytokines and TNF-α, IL-1β, and IL-6.	[157]
		Human ARPE-19 cells	Activated Nrf2 signaling cascade.	[160]
		RPE cells	Increased the expression of Nrf2/HO-1, ERK1/2, and reduced ROS-induced apoptosis.	[161]
		Streptozotocin (STZ)-induced diabetic rats	Improved histopathologic parameters, markers of oxidative stress, and plasma glucose. Regulatory effect on Nfr2 pathway activation. Inhibition of the AGE-RAGE pathway and extracellular matrix (ECM)-receptor interaction in the retina	[162]

**Table 3:** Primary outcomes of the studies testing the use of quercetin in diabetic retinopathy.

Compound	Food Source	Effects on the Retina		
		Experimental Model	Effects	Reference
Quercetin	Teas (from *C. sinensis*), onions, apples, and berries.	ARPE-19 cells hyperglycemic medium	Decreased inflammatory markers, phosphorylation of MAPKs (ERK1/2, p38, and JNK1/2), and cAMP response element-binding protein (CREB). Activation of transcription factor 2 (ATF2), c-Jun, and nuclear factor-B kinase (IKK)/NFκ-B.	[186]
		Intraperitoneal injection in diabetic rats	Reduced the number of apoptotic cells and partially improved the retinal histopathology (decreased the thickness of the inner and outer nuclear layer, decreasing the distance between the inner and outer limiting membrane). Increased the number of ganglion cells. Decreased pro-inflammatory cytokines, production of pro-angiogenic factors and soluble adhesion proteins, VEGF, and sICAM-1.	[187]
		HRMEC incubated in a hyperglycemic medium	Inhibits the activation of angio-genic, inflammatory, and autophagic mechanisms.	[188]
		STZ diabetic rats	Improved histology in the retina (less edema and cell vacuolization). Reduced expression of matrix metalloproteinase-9 and VEGF proteins.	[189]
		Diabetic rats	Increased retinal BDNF, NGF, TrkB, and phosphorylation of Akt. Prevented apoptosis, decreased cytochrome c and caspase-3, and increased anti-apoptotic protein Bcl-2.	[190]
		Human ARPE-19 cells incubated with NaIO_3_ (induce age-related macular degeneration-like retinal pathology)	Inhibited the PI3K/Akt pathway and stimulated Bcl-2 production.	[191]

**Table 4:** Primary outcomes of the studies testing the use of resveratrol in diabetic retinopathy.

Compound	Health Benefits	Effects on the Retina		
		Experimental Model	Effects	Reference
Resveratrol	Anti-tumor, anti-aging, cardioprotective, neuroprotective, and antioxidant.	Diabetic rat retina	Inhibited NF-kB activity. Reversed hyperglycemia Promoted vascular enlargement in the ganglion cell layer and increased VEGF. Inhibited the transcription factor levels of inflammatory and cell death proteins.	[200,201,204]
		Diabetic rat retina and Muller cells culture	Increased microRNA 29b (associated with inhibition of SP1, activated caspase 3, and Bax) and increased Bcl-2.	[202]
		BRECs incubated hyperglycemic medium	Prevents the degradation of AMPK, SIRT-1, and PGC-1α.	[206]
		Peripheral blood mononuclear cell cultures from patients with proliferative diabetic retinopathy	Increased SIRT-1 Levels and Decreased Proinflammatory Cytokine	[207]
		HRECs incubated in a hyperglycemic medium	Inhibited PKC activation leads to the non-production of ROS, thus inhibiting the phenotypic change from endothelial to mesenchymal cells.	[209]
